# The Effect of Dynamic Food Labels with Real-Time Feedback on Diet Quality: Results from a Randomized Controlled Trial

**DOI:** 10.3390/nu12072158

**Published:** 2020-07-20

**Authors:** Soye Shin, Rob M. van Dam, Eric A. Finkelstein

**Affiliations:** 1 Program in Health Services and Systems Research, Duke-NUS Medical School, Singapore 169857, Singapore; syshin@duke-nus.edu.sg; 2 Saw Swee Hock School of Public Health, National University of Singapore, Singapore 117549, Singapore; rob.van.dam@nus.edu.sg

**Keywords:** front-of-pack labeling, nutrition labeling, diet quality, dynamic labels, real-time feedback, online grocery store, Nutri-Score

## Abstract

The rising prevalence of non-communicable diseases has brought attention to the importance of consuming a healthy diet. One strategy to improve diet quality is through front-of-pack (FOP) nutrition labels. Taking advantage of an online grocery store, we allowed consumers to choose the FOP labels they preferred, and combined this information with real-time feedback on the overall nutritional quality of the shopping basket. We hypothesized that these dynamic food labels with real-time feedback (DFLF) would improve nutritional quality of food purchases. This trial followed a two-arm (no-label control and DFLF) crossover design with 125 participants exposed to each condition once in random order via an online grocery store. A first difference regression model allowed for estimating the unbiased effect of the DFLF on diet quality, measured by the weighted average Nutri-Score (ranging 1 to 5) per serving (primary) and changes in select nutrients and calories. The mean weighted Nutri-Score was 0.4 (12.6%) higher in the DFLF arm (CI: [0.2, 0.6]) relative to the control. The DFLF also decreased the amount of sugar per serving by 0.9 g (CI: [−1.7, −0.0]) and total sugar per shop by 169.5 g (CI: [−284.5, −54.5]). The DFLF features significantly improved nutrition quality relative to no labelling, as measured by average Nutri-Score values. These results shed light on the considerable potential of the online shopping environment to improve diet quality through customization and real time feedback.

## 1. Introduction

The global obesity and non-communicable diseases (NCDs) epidemics have brought attention to the importance of consuming a healthy diet. An extensive body of literature has shown that low-quality diets contribute to excessive weight gain [1], obesity [2], and major NCDs that include type 2 diabetes [3,4], cardiovascular diseases [5,6], nonalcoholic fatty liver disease [4], and several cancers [7]. Diets lacking essential vitamins and nutrients can also lead to health conditions related to malnutrition, a growing problem among the elderly [8].

Despite the well-established link between diet quality and health, consumers often fail to make healthier food choices at the point of purchase. Many are unaware of the nutritional quality of foods purchased, even when this information is available on the nutrition facts panel (NFP) on the back of the products [9,10,11]. This is because the NFP is not salient and the many dimensions make it difficult to understand for the average consumer [12,13,14,15]. As a result, it is often ignored [16].

One strategy to overcome these barriers is through the implementation of salient and easy to comprehend front-of-pack (FOP) nutrition labels. FOP labels can generally be classified as reductive or interpretative. Reductive labels present information without interpretation, such as presenting calories per serving. Interpretive labels use the underlying information to convey a message to consumers as to the underlying health of the product in the dimensions considered. The color-coded Multiple Traffic Light (MTL) label used in the UK, warning labels now used in Chile and Israel, Singapore’s Healthier Choice logo, and France’s Nutri-Score labels are examples of interpretive labels. Interpretive labels are both more salient and easier to understand than reductive labels [17], yet both FOP strategies have been shown to help consumers make healthier purchases [10,18,19,20,21,22,23,24,25,26].

Although many FOP labels have been shown to be effective, on average, it is likely that greater effectiveness can be realized through customization and real time feedback. That results because consumers have different preferences for nutritional information. For example, some may wish to focus on sodium reduction, others on calories, and others on ensuring that they get enough of key nutrients, such as calcium. The online shopping environment provides the opportunity to customize the labels based on consumer preferences and to offer real-time feedback on the nutritional quality of the shopping basket. The aim of the study is to test the effectiveness of such a strategy on the nutritional quality of food purchases.

Using an experimental online grocery store with the chance of real food purchase and delivery, we designed the dynamic food labels with real-time feedback (DFLF) tools for consumers to use at any time during their shop. Specifically, we allowed consumers to choose between any of seven FOP labels at the click of a button. We also provided behavioral nudges designed to increase diet quality regardless of which label was chosen and real-time feedback of the healthiness of the chosen basket.

We hypothesized that, compared to the control:

- **Hypothesis 1:***Nutritional quality of food purchases measured by weighted (by the number of servings) average Nutri-Score per serving (primary outcome) would be higher in the DFLF arm*.
- **Hypothesis 2:***Average calories, sugar, sodium, total fats, and saturated fat per serving, and total calories and total sugar per shop would be lower in the DFLF arm*.
- **Hypothesis 3:***Calories per dollar spent would be lower in the DFLF arm*.

We tested these hypotheses using a two-arm randomized control crossover experiment among Singaporean shoppers using a fully functional online grocery store where real purchase chances were imposed. As some evidence shows that hunger and mood influence impulse purchases [27,28], we also explored whether these factors moderate the results of the primary measure of nutritional quality.

## 2. Experimental Design and Methods

### 2.1. Online Grocery Store

**Figure 1:**
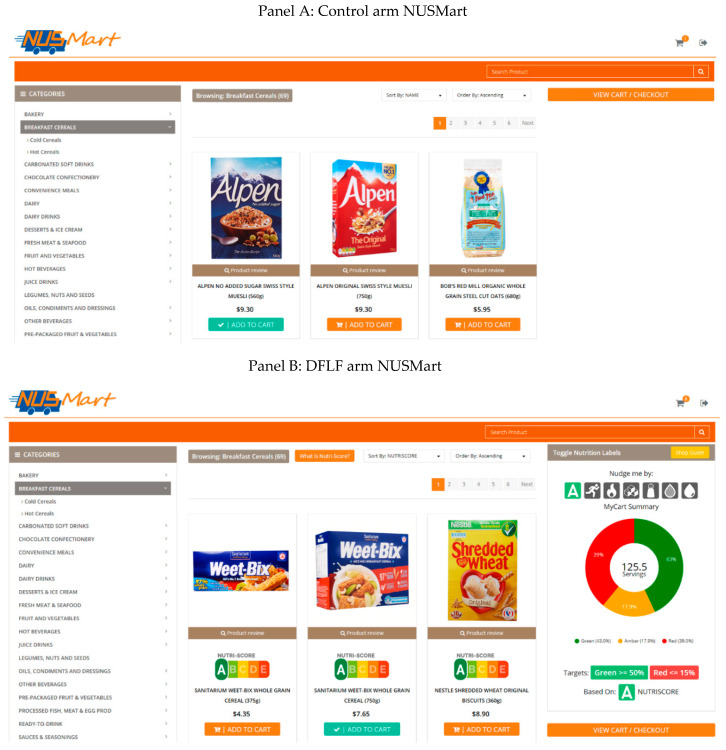
Screenshots of the two versions of the NUSMart webpage: (**A**) control arm and (**B**) dynamic food labels with real-time feedback (DFLF) arm.

We used an experimental online grocery store developed for research purposes, called NUSMart, for this study (Figure 1). NUSMart was designed to mirror a commercial web-based grocery store but to be highly flexible to test various hypotheses regarding how to improve grocery shopping behavior. Participants are able to add and remove products to and from their online grocery cart and see the effects on total costs. In partnership with a local online retailer, we were able to deliver the products purchased by the participants to their homes for a subset of purchases. At the point of enrollment, we informed participants that the probability of a real purchase was 50% for every shopping trip. More study details are in Section 2.3.

At the time of this study, the store contained 3381 food and beverage (F&B) products commonly purchased in Singaporean supermarkets. All products shown had pictures of the item, retail prices, product descriptions, and nutritional information available via click-through. Foods were classified into one of 27 categories, and then by subcategories within the broader category. We list the category–subcategory pairs used for NUSMart in Appendix A Table A1 and also provide additional images of the NUSMart interface.

### 2.2. Features of the Dynamic Food Labels with Real-time Feedback (DFLF) Arm

The DFLF consists of the following features: The seven FOP labels include Nutri-Score as a summary measure of overall nutritional quality; calories in the form of a physical activity equivalents (PAE) label; calories per serving; and per serving values of four nutrients, including sugar, sodium, saturated fat, and total fat. We also provided behavioral nudges designed to increase the nutritional quality of food purchases regardless of which label was chosen. The first nudge was the ability to reorder products from most to least healthy, as food repositioning has been shown to nudge consumers toward healthier purchases [29,30]. We also provided real-time feedback of the healthiness of the chosen basket with a traffic light color-coded pie chart and a recommended target for a healthy grocery basket. The intuitive and real time use of the traffic light colors and approach increases salience [31] and decreases cognitive burden [19,32], which enhances the impact on healthier food choices [33,34]. Lastly, before checkout, consumers were able to see an overall healthiness summary of their final shopping baskets across the seven nutrient labels at the product level. This provides a timely opportunity to make improvements in diet quality prior to checkout. We explain each DFLF component in detail below.

#### 2.2.1. Dynamic Displays of FOP Labels on Product Listings

The DFLF allowed participants to customize the display by choosing one of the following 7 FOP labels:

(1) Nutri-Score (NS) which is a color-coded index of overall diet quality ranging from A (healthiest) to E (least healthy) based on the British Food Standard Agency Nutrient Profiling System. To assign an NS grade (A to E) to each product, we applied the standard Nutri-Score algorithm [35,36,37] for food products but applied a modified algorithm for beverages based on the Singapore Health Promotion Board Proprietary Scoring System, which focuses more on calories and sugar content than the original algorithm.
(2) Calorie information in the form of a physical activity equivalents (PAE) label. This label shows the amount of time required for participants to burn off the calories in a single serving by jogging. There is evidence that showing calorie information in this form increases salience and is thus more effective than simply showing calories [38].
(3) Calories per serving and percentage of daily recommended intake.
(4) Sugar content per serving and percentage of daily recommended intake.
(5) Sodium content per serving and percentage of daily recommended intake.
(6) Saturated fat content per serving and percentage of daily recommended intake.
(7) Total fat content per serving and percentage of daily recommended intake.

The daily recommended values of nutrients came from the multiple traffic light (MTL) algorithm created by the United Kingdom Food Standards Agency [39].

**Figure 2:**
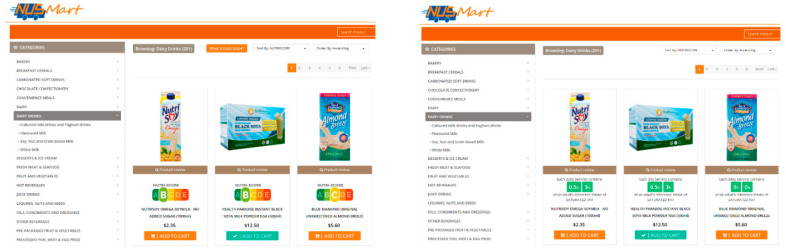
A screenshot of a dairy drink product listing with two different types of labels. (**Left**: Nutri-Score label. **Right**: saturated fat label).

We displayed seven buttons of simple icons representing these seven nutrient labels (Figure 1B top right) on the right panel of the web page. The default FOP label was Nutri-Score. Figure 2 shows how nutrient information on the same dairy drink product listing differed based on which nutrient label is selected. As the figure shows, nutritional information right below each product picture was color-coded for healthiness. The kcal and nutrient-specific food labels used three traffic light colors (green, healthier; amber, intermediate; red, less healthy) based on the MTL scheme, whereas the Nutri-Score used similar colors but with finer gradations for the five healthiness scales (A to E).

Using those seven icons, participants were able to switch between the displays of different nutrient labels on product listings at any point during the shopping experience. By toggling between the labels, shoppers were able to conduct quick evaluations of products according to specific nutritional contents.

We also provided a “Sort by” button on the top of the store where shoppers could choose any of the included food labels and sort products within the category using that label from most to least healthy within the food category by the click of a button. For instance, participants could reorder beverages from lowest to highest sugar or calories per serving. 

#### 2.2.2. Real-Time Feedback on Grocery Carts

The DFLF also provided a live visual indicator of the healthiness of the shopper’s current basket as a pie chart, called “MyCart Summary” (Figure 1B, far right panel), showing the proportion of healthy/less healthy products by servings for the selected nutrient attribute with the three MTL colors. For the Nutri-Score which had five colors, we aggregated A and B to green (healthier) and D and E to red (least healthy) while presenting C as amber (in the middle). This way, as shoppers added a product to their baskets, they had visual real-time feedback on how their latest addition contributed to their total basket healthiness via the pie chart. The MyCart Summary also provided a target for a healthy grocery basket (at least 50% green and a maximum of 15% red) that participants could use as a healthy reference to target.

#### 2.2.3. The Healthiness Summary of Final Grocery Carts

**Figure 3:**
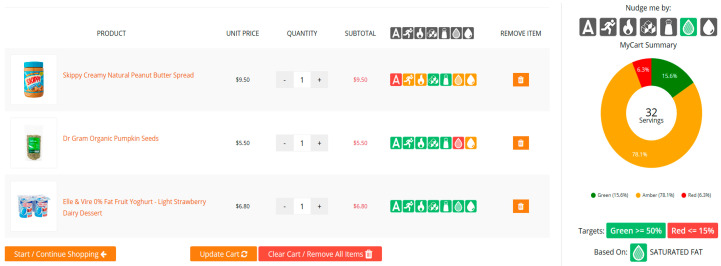
A screenshot of a shopping basket summary along with MyCart Summary.

The last function provided an overall healthiness summary of participants’ shopping baskets before checkout. Figure 3 presents a screenshot of a shopping basket summary page. For each product a participant chose to buy, the healthiness of the product was evaluated by the seven different food labels with the MTL colors. This may be helpful to consumers as it provides a holistic view of the products and a final chance to switch to healthier options.

### 2.3. Experimental Design and Data Collection

**Figure 4:**
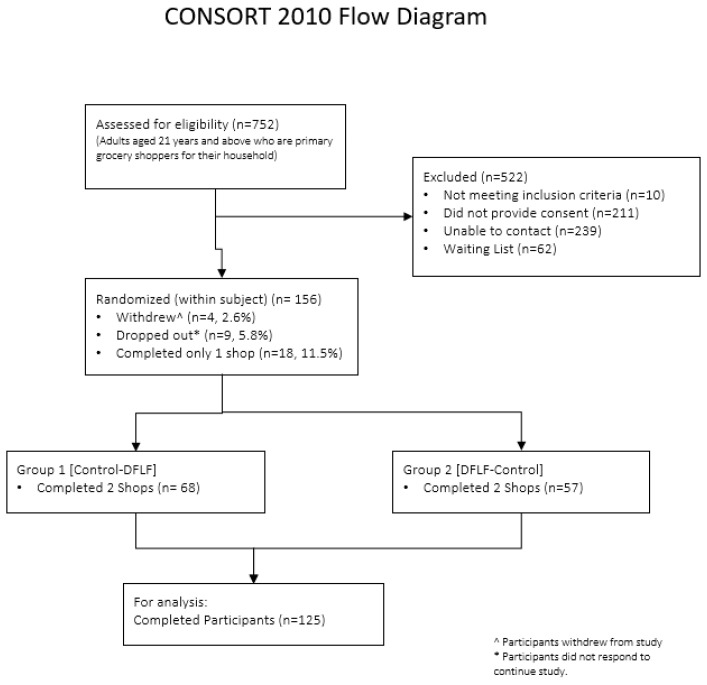
CONSORT flow diagram for participation recruitment and randomization.

We conducted the study in Singapore using a 2 × 2 crossover randomized controlled trial design with two versions of NUSMart (control and DFLF arms). With the crossover design, all participants were exposed once to each of the two shopping conditions in random order that was predetermined via random permuted blocks of size two with equal allocation by a computer program. While participants were made aware that there were two versions of NUSMart, the exact details of each version were not revealed to ensure validity of results. The sample size was estimated based on the ability to detect a standard effect size of 0.29 in the weighted average of Nutri-Score between the two arms with power of 0.80 and a significance level of 0.05. Including a 10% attrition rate based on previous studies using NUSMart, we estimated that the required sample size was 136. To ensure sufficient power, we recruited 156 participants among whom 125 participants completed two shopping trips. We present participant flow and randomization in Figure 4.

Participants were recruited via online advertisements from October to December 2019. Prospective participants were directed from recruitment advertisements to the NUSMart website (https://nusmart.duke-nus.edu.sg/SSA) and asked to complete an online screener to determine their eligibility. Potential participants were eligible to participate if they were Singapore residents, aged 21 years or above, and the primary grocery shopper for their household. At point of enrollment, we informed them that they were required to complete two weekly shopping trips as they normally do in a typical grocery shopping experience with a minimum spend of 50 Singapore Dollars (SGD) and a maximum spend of 250 SGD. They were also told that for every shopping trip they had a 50% chance of having to purchase the selected groceries prior to checkout. The chance of real purchases was also randomized at point of enrollment when the randomization of the order of the two versions of NUSMart was made. It is worth noting that we did not impose the real purchase requirement for every order due to the heavy logistical burden of processing the payment and delivery of the orders. However, we believe that imposing a real purchase chance ensures that participants take the shopping exercise seriously and the data highly likely reflects participants’ actual shopping behavior.

Those interested and eligible were asked to complete the following components online: (1) a registration form containing their name, mobile phone number, and email address; (2) an online consent form; and after online consent (3) a baseline survey collecting their demographic characteristics, including age, gender, ethnicity, and monthly household income. Upon completion of all forms, the website created the participant’s account and unique participant identification number (PID) for use throughout the study. Participants then received automated emails with their unique login details and were asked to logon to the NUSMart online grocery store to complete the two shopping tasks. Prior to the shopping trip using the NUSMart with DFLF, there was a pop-up window briefly explaining how to use the four DFLF features with seven nutrient labels.

Upon completion of each shopping trip, participants were asked their mood and hunger at the time of placing their order. The mood level took the values 1 (very unhappy) to 5 (very happy). The hunger level took the values of 1 (not at all hungry) to 10 (extremely hungry). On their final shop (the second trip), participants were also asked to complete a post-study survey that included feedback queries on the study and their experience with the DFLF. The post-study survey, which included questions on current mood and hunger, was also conducted online. Once participants completed every element of the study, they received a reimbursement of 50 SGD in the form of an e-voucher as compensation for their participation.

The study was conducted according to the guidelines laid down in the Declaration of Helsinki and all procedures were approved by the National University of Singapore Institutional Review Board Reference Code: S-19-154. Informed consent was obtained from all subjects. The trial was registered on the American Economic Association’s registry for randomized controlled trial, RCT ID: AEARCTR-0004520; registered 08 August 2019.

### 2.4. Statistical Analysis

#### 2.4.1. Outcome Variables

To test the effect of the DFLF on the nutritional quality of shoppers’ grocery baskets, we generated the following outcome variables. The primary outcome variable was the average Nutri-Score per serving weighted by the number of servings. Specifically, for each grocery order we recoded Nutri-Score from A=5 (the highest nutritional quality) to E=1 (the lowest nutritional quality) for each product and then calculated a weighted (by the number of servings) average score over numeric values of all products in the basket. We used the Nutri-Score per serving as the primary variable because it was the most direct measure of the effects of our intervention given that the default label in the DFLF arm was the Nutri-Score.

The secondary measures of nutritional quality included total calories and sugar purchased, and average per serving of calories, sugar, sodium, total fat, and saturated fat. Considering that calories per dollar from healthier foods tend to be relatively lower compared to less healthy foods, we also calculated kcal per dollar spent for each shopping episode.

#### 2.4.2. Model

We tested the hypotheses by estimating the following first difference regression via ordinary least squares (OLS), with the difference being (Y in the DFLF arm) – (Y in the control arm) for participant j:

$$\Delta Y_{j}=\alpha +\epsilon_{j}$$

where $\Delta Y_{j}\;$ is the first differenced outcome observed for participant j, α is a constant term as a result of the first difference of an indicator variable for the DFLF intervention for participant j, and $\epsilon_{j}$ is the first difference-within-an-individual random measurement error. The direction of α, the coefficient of the constant term, was our primary interest. For Hypothesis 1 (a weighted average NS per serving is higher in the DFLF arm), we expected a positive coefficient, whereas negative coefficients both for Hypothesis 2 (the total and average nutrients to limit are lower in the DFLF arm) and Hypothesis 3 (calorie per dollar is lower in the DFLF arm) were expected. We ran the same regression for beverages only.

To test the heterogeneous effects of the DFLF by mood and hunger which vary by each shopping trip, we included the within-individual first differenced mood and hunger in the main estimating model:

$$\Delta Y_{j}=\alpha +\mu^{\prime }\Delta M_{j}+\epsilon_{j}$$

where $\Delta M_{j}$ is a vector of the first differenced moderating factors of interest. The variable can take on values of −4 to 4 for mood and −9 to 9 for hunger.

## 3. Results

### 3.1. Descriptive Analysis of Sample

Table 1 presents the descriptive statistics for the study population. The participants were largely of Chinese ethnicity (90%) and female (72%). The age of participants ranged from 21 to 66 years with the mean of 36 (SD = 9.30). Participants were generally highly educated with 78 percent achieving the educational attainment of “University degree or above.” The portion of the participants whose household income was “$10,000 and above” was 27% and the mean household size was 3.41. The average BMI was 23.14 kg/$m^{2}$ (SD = 4.18) and 14 percent had diabetes.

### 3.2. The Effect of the DFLF on Nutritional Quality of Food Purchases

Table 2 shows the effects of the DFLF intervention on different measures of nutritional quality of the food and beverage purchases. Specifically, for each outcome variable, we report the unadjusted mean value for the DFLF arm (column 1) and the control arm (column 2), with their between-arm difference (which is equivalent to the coefficient on the constant term; α) along with the 95% confidence interval (column 3).

The mean weighted average Nutri-Score per serving was 3.26 in the control. The average Nutri-Score increased by 0.41 (12.6%) to 3.67 in the DFLF arm (CI: [0.24, 0.58]) compared to the control. This result is consistent with Hypothesis 1: that the DFLF features improve nutritional quality. We also compared the proportions of green (Nutri-Score A&B), amber (Nutri-Score C), and red (Nutri-Score D&E) products between the control and the DFLF arm. Whereas in the control the percentages were 56%, 17%, and 27%, respectively, these percentages improved to 66%, 16%, and 18% in DFLF. Among the nutrient-specific measures, we found that the DFLF significantly decreased the amount of sugar per serving by 0.85 g (CI: [−1.70, −0.00]) and total sugar per shop by 169.53 g (CI: [−284.53, −54.53]) relative to the control (6.12 g and 593.86 g, respectively). For other nutrients, we did not find significant effects of the DFLF, although the signs of the coefficients were as predicted except for average kcal per serving.

Table 3 presents the effects of the DFLF intervention on the nutritional quality of beverages. When limited to beverages only, we found even stronger effects of the DFLF on the weighted average Nutri-Score (the relative change) and sugar per serving. The mean weighted average Nutri-Score per serving was 1.81 in the control. The average Nutri-Score increased by 0.38 (21%) to 2.18 in the DFLF arm (CI: [0.08, 0.67]). Whereas the percentages of green (Nutri-Score A&B), amber (Nutri-Score C), and red (Nutri-Score D&E) beverage products were 14%, 16%, and 70% in the control, respectively, these percentages improved to 23%, 16%, and 61% in DFLF. The amounts of average sugar per serving and total sugar per shop through beverages were reduced by 3.21 g (CI: [−6.02, −0.39]) and 96.6 g (CI: [−178.8, −14.39]), respectively. We again did not find a reduction in calories in the DFLF arm relative to the control.

For the primary outcome, we also report the results of the first difference model for possible heterogeneous effects by mood and hunger—one for food and beverages (F&B) and the other for beverages only. Results appear in columns 1 and 2 of Table 4. We found that for F&B, Nutri-Score increased by 0.22 more in the DFLF arm relative to the control when a shopper’s happiness increased by one unit. However, this coefficient was only significant at the 10% level. For beverages only, we could not reject the hypothesis that mood and hunger did not affect food choices.

## 4. Discussion

In this study, we devised a novel intervention with dynamic FOP labels and other nudges and assessed its effects on the nutritional quality of food purchases in an online grocery store built for research purposes. We showed that the intervention improved the nutritional quality of food purchases measured by a higher weighted Nutri-Score per serving and lower sugar content of purchased products. Not only were the features employed in our study effective, our post-study survey revealed that most participants found the features user-friendly (mean value: 3.31 out of 4) and useful (mean value: 3.26 out of 4), meaning if made available for public use there will likely be significant interest in such a tool.

To our knowledge, this is the first trial to estimate the effects of dynamic food labels. Although it is difficult to compare across studies, the effects of our dynamic labeling intervention appear larger than those observed for static labels in previous intervention studies. For example, in a review article [40], Croker et al. (2020) showed that static labels reduced sugar purchased from 0.20g to 0.84g per 100g for all foods and beverages and by 0.33g per 100g for beverages only. When our sugar reduction per serving is converted to per 100g, we find larger reductions of 0.89g for all foods and beverages and 1.81g for beverages only. Although this finding is suggestive, future studies should rigorously compare dynamic to static labels to quantify the increased of the dynamic labelling approach.

This study is novel in that it is the first to explore the effectiveness of dynamic food labels and other features aimed to nudge consumers toward purchasing healthier food products using an online grocery store with real purchases. Although we cannot conclude based on our current study which features were most effective, or whether other features may increase effectiveness, these significant effects provide a future direction of food labelling strategies and suggest that diet quality can be further improved by taking advantage of an online shopping environment. This is important not just in Singapore but worldwide, given that poor diet quality is a worldwide phenomenon, and partly due to Covid-19, consumers are quickly transitioning from brick and mortar stores to online shopping [41].

It is worth noting that in addition to improvements in overall nutritional quality, DFLF significantly reduced sugar intake but had no significant effects on calories or other nutrients. This likely resulted because participants were likely to have shopped with the default FOP label, Nutri-Score (NS), which is most highly correlated with sugar (F&B; −0.45, significant; beverages; −0.46, significant) and least correlated with calories (F&B; −0.05, insignificant; beverages; −0.35, significant). We chose NS as the default label because it is the most holistic measure of nutritional quality and we expected many shoppers to stay on the default option [42,43,44]. Due to data limitations, we were unable to analyze consumers’ DFLF use patterns, such as how often they switched between labels. This will be the subject of future research. Lastly, we could not reject the null effect of mood and hunger: that mood and hunger do not influence food choices. Although this may be due to a lack of power, the influences of these factors are likely attenuated in the online shopping environment as there is a greater time lag between purchase and consumption [45].

Our study has several strengths, including a rigorous randomized crossover experimental design and a fully-functional online grocery store which is likely to have led to more realistic shopping behaviors by imposing actual payment and delivery of orders for a subset of shops. However, the study has several limitations. Coupled with the data unavailability to examine consumers’ DFLF use patterns, another limitation is that results were based on a single shopping episode, and thus may not be sustained over multiple shops. Testing the effects of DFLF on nutritional quality in the real world over repeated shops should be an area of future research.

## 5. Conclusions

Relative to a standard online grocery store, the DFLF resulted in significant improvements in nutritional quality of food purchases, measured by a higher weighted average Nutri-Score value per serving and lower sugar content of purchased products. Overall, the results shed light on the considerable potential of the online shopping environment to improve diet quality through customization and real time feedback.

## Figures and Tables

**Table 1:** Participant summary statistics.

Variable	Mean	(S.D.)
Age (years)	36.17	(9.30)
Chinese (1/100%)	0.93	(0.26)
Female (1/100%)	0.72	(0.45)
Married (1/100%)	0.53	(0.50)
Education (University and above) (1/100%)	0.78	(0.42)
Income ($10,000 and above) (1/100%)	0.27	(0.45)
Household size	3.41	(1.55)
BMI (kg/$m^{2}$)	23.14	(4.18)
Diabetes (1/100%)	0.14	(0.35)
Overweight/Obesity (1/100%)	0.18	(0.39)
Observations (N)	125	125

**Table 2:** The effects of the dynamic food labels with real-time feedback (DFLF) on the nutritional quality of purchased foods and beverages.

	(1) Control	(2) DFLF	(3) DFLF-Control	
Outcome Variable	Mean		Difference	[95% CI]
Weighted Nutri-Score per serving	3.26	3.67	0.41 ***	[0.24, 0.58]
Total kcal per shop (in 1000s)	14.64	13.10	−1.54	[−3.70, 0.61]
Average kcal per serving	141.15	142.68	1.53	[−10.0, 13.1]
Total sugar (g) per shop	593.86	424.33	−169.53 ***	[−284.53, −54.53]
Average Sugar (g) per serving	6.12	5.26	−0.85 **	[−1.70, −0.00]
Average Sodium(mg) per serving	163.77	142.84	−20.93	[−59.52, 17.65]
Average Total Fat (g) per serving	5.33	5.11	−0.21	[−1.05, 0.63]
Average Saturated Fat (g) per serving	1.69	1.62	−0.065	[−0.39, 0.26]
kcal per dollar (SGD) spent	243.35	235.01	−8.35	[−45.1, 28.4]
Observations (N)	125	125		

Note: 95% confidence intervals are in brackets. ** *p* < 0.05, *** *p* < 0.01.

**Table 3:** The effects of the dynamic food labels with real-time feedback (DFLF) on the nutritional quality of purchased beverages.

	(1) Control	(2) DFLF	(3) DFLF-Control	
Outcome Variable	Mean		Difference	[95% CI]
Weighted Nutri-Score per serving	1.81	2.18	0.38 **	[0.08,0.67]
Total kcal per shop (in 1000s)	2.30	1.98	−0.33	[−0.92,0.27]
Average kcal per serving	111.37	118.00	6.62	[−30.10, 43.35]
Total sugar (g) per shop	325.74	229.12	−96.62 **	[−178.85, −14.39]
Average Sugar (g) per serving	15.19	11.98	−3.21 **	[−6.02, −0.39]
Average Sodium (mg) per serving	61.21	76.55	15.35	[−6.77, 37.47]
Average Total Fat (g) per serving	2.50	3.64	1.14	[−0.61, 2.89]
Average Saturated Fat (g) per serving	1.46	2.12	0.66	[−0.42, 1.75]
kcal per dollar (SGD) spent	36.96	35.24	−1.72	[−12.60, 9.16]
Observations (N)	66	66		

Note: 95% confidence intervals are in brackets. ** *p* < 0.05.

**Table 4:** The heterogeneous effects of the dynamic food labels with real-time feedback (DFLF) on nutritional quality by hunger and mood.

	(1) Food and Beverages	(2) Beverages Only
	NS per Serving (/s)	NS per Serving(/s)
constant	0.45 ***	0.47 ***
	[0.28, 0.63]	[0.19, 0.74]
Δhunger	0.01	0.01
	[−0.06, 0.08]	[−0.10, 0.12]
Δmood	0.22 *	0.20
	[−0.02, 0.46]	[−0.15, 0.55]
Observations	121	64

Notes: 95% confidence intervals are in brackets. * *p* < 0.1, *** *p* <0.01.

**Figure A1:**
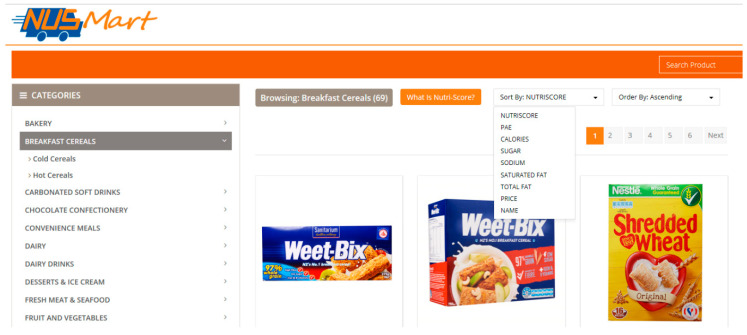
A screenshot of the product-reordering function by food labels. Note: The default food label was Nutri-Score.

**Figure A2:**
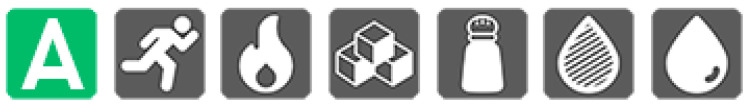
Seven icons representing the seven different nutrient labels, respectively. Note: The active icon affecting the DFLF functions is colored in green. The active button in Figure A2 represents the Nutri-Score label.

## Appendix A

**Table A1:** The list of categories and subcategories on NUSMart.

ID	Category	Subcategory
1	Bakery	Baking Ingredients & Mixes
2		Biscuits, Crackers & Cookies
3		Bread & Bread Products
4		Cakes, Pastries & Sweet Goods
5	Breakfast Cereals	Cold Cereals
6		Hot Cereals
7	Carbonated Soft Drinks	Carbonated Soft Drinks
8		Chocolate Bars
9		Chocolate Tablets
10		Festive Chocolate
11		Individual Bar & Pack Chocolate
12		Non-Individually Wrapped Chocolate
13	Convenience meals	Instant Noodles
14		Instant Pasta
15		Instant Rice
16		Meal Kits
17		Pastry Dishes
18		Pizzas
19		Prepared Meals
20	Dairy	Butter, Spreads and Margarine
21		Cheese Spreads
22		Creamer
23		Fresh Cheese & Cream Cheese
24		Fresh Cream
25		Hard Cheese & Semi-hard Cheese
26		Processed Cheese
27		Soft Cheese & Semi-soft Cheese
28		Yogurt
29	Dairy Drinks	Cultured milk drinks and Yoghurt drinks
30		Flavoured Milk
31		Soy, Nut and Grain-based Milk
32		White Milk
33	Desserts & Ice Cream	Chilled Desserts
34		Frozen Desserts
35		Ice Cream
36		Jellies and Puddings
37		Shelf-Stable Desserts
38	Fresh Meat & Seafood	Fresh Fish & Seafood
39		Fresh Meat
40		Frozen Fish & Seafood
41		Frozen Meat
42	Fruit and Vegetables	Fresh Fruits
43		Fresh Vegetable
44		Frozen Fruits
45	Hot Beverages	Coffee
46		Malt & Other Hot Beverages
47		Tea
48	Juice Drinks	Fruit/Flavoured Still Drinks
49		Juice
50		Nectars
51	Legumes, Nuts and Seeds	Legumes, Nuts and Seeds
52	Oils, Condiments and Dressings	Dressings & Vinegar
53		Mayonnaise
54		Oils
55	Other Beverages	Beverage Concentrates
56		Beverage Mixes
57		Meal Replacements & Other Drinks
58	Pre-Packaged Fruit & Vegetables	Canned Fruit
59		Canned Vegetables
60		Frozen Vegetables
61	Processed Fish, Meat & Egg Prod	Eggs & Egg Products
62		Fish Products
63		Meat Products
64		Poultry Products
65	Ready-To-Drink	Other RTDs
66		RTD (Iced) Coffee
67		RTD (Iced) Tea
68	Sauces & Seasonings	Cooking Sauces
69		Other Sauces & Seasonings
70		Pasta Sauces
71		Pickled Condiments
72		Seasonings
73		Stocks
74		Table Sauces
75	Snacks	Chips, Crisps and Crackers
76		Corn-Based Snacks
77		Fruit Snacks
78		Hors d’oeuvres/Canapes
79		Meat Snacks
80		Popcorn
81		Snack Mixes
82		Snack/Cereal/Energy Bars
83		Vegetable Snacks
84	Soup	Dry Soup
85		Wet Soup
86	Sports & Energy Drinks	Energy Drinks
87		Sports Drinks
88	Spreads	Caramel & Cream Spreads
89		Chocolate Spreads
90		Dips
91		Honey
92		Jam and Marmalade
93		Kaya
94		Nut Spreads
95		Savoury Spreads
96		Syrups
97		Chilled Meals
98		Noodles
99		Pasta
100		Rice
101		Stuffing, Polenta & Other Side Dishes
102	Sugar & Gum Confectionery	Chewy Sweets and Marshmallows
103		Lollipops and Hard Candy
104		Mints and Lozenges
105		Pastilles, Gums, Jellies & Chews
106		Toffees, Caramels & Nougat
107	Sweeteners & Sugar	Artificial Sweeteners
108		Creamer
109		Natural Sweeteners
110		Sugar
111		Sugar Cubes and Crystals
112	Vegetarian Meat & Seafood	Plant-Based Meat and Seafood Alternatives
